# Who Wants Wiley Fired?

**Published:** 1911-09

**Authors:** 


### Who Wants Wiley Fired?

Who wants Wiley fired?
"I," says the can of nearly tea.
"Just look what he did to me.
He or I must be retired.
So I want Wiley fired."

Who wants Wiley fired?
"I," says the case of almost cheese.
"Once I lived a life of ease.
But now this fellow makes me tired.
So I want. Wiley fired."

Who wants Wiley fired?
"I," says the ham that's acid cured.
"This buttin' in can't be endured.
The wonder is that he was hired.
Sure, I want Wiley fired."

Who wants Wiley fired?
"I," says the masquerading jam,
A product he has tried to damn.
" 'Get rid of him!' is what I wired.
Yes, I want Wiley fired."

Who wants Wiley fired?
Why, all the bogus foods and drugs,
And all the germs and microbe bugs.
There's nothing quite so much desired
As to see Wiley fired.—C. W. S., in *N. Y. World.*

